# Reducing interrogative suggestibility: The role of self-affirmation and positive feedback

**DOI:** 10.1371/journal.pone.0236088

**Published:** 2020-07-21

**Authors:** Malwina Szpitalak, Romuald Polczyk

**Affiliations:** Institute of Psychology, Jagiellonian University, Kraków, Poland; Ghent University, BELGIUM

## Abstract

Interrogative suggestibility, as measured with Gudjonsson Suggestibility Scales, consists of an individual's tendency to yield to misleading questions (Yield) and to change answers after negative feedback (Shift). This study aimed to determine whether reinforced self-affirmation (RSA), a technique that aims to boost self-confidence in order to increase the tendency to rely on one’s own memory instead of external cues, can reduce interrogative suggestibility. RSA consists of self-affirmation induced by means of writing down one’s greatest achievements in life and of manipulated positive feedback. The efficacy of two kinds of positive feedback was explored. Shift was reduced by positive feedback relating both to memory and to the feeling that a person is very independent in their judgements, while only feedback related to memory reduced Yield. The results are discussed in terms of the different mechanisms underlying Yield and Shift. Inducing independence of judgements might not have been effective in the case of Yield because to some extent it taps opinions but not the quality of a cognitive process such as memory. An individual may believe in their own opinions and views but still be unsure about the quality of their own memory.

## Introduction

Eyewitness testimony is very important in court cases [1, 2]. Nevertheless, eyewitnesses are prone to make mistakes and testify incorrectly despite the fact they do not mean to lie [3] and it is now well established that human memory is prone to distortions [e.g. 4–9]. An important cause for such distortions may be the fact that an interviewed person could be uncertain of their memories and answer wrongly in accordance to suggestion or misinformation, even though their memory is correct [10, 11]. What seems important is that forensic psychologists should know some methods that would be helpful in immunizing witnesses against misinformation and enhancing the quality of their testimony. The aim of this paper is to present results concerning one of such methods, applied in the context of interrogative suggestibility [12]. We first present the concept of interrogative suggestibility, then review the existing methods for reducing it, and present the rationale for our method aiming to reduce it.

Interrogative suggestibility (IS) is defined as ‘the extent to which, within a closed social interaction, people come to accept messages communicated during formal questioning, as a result of which their subsequent behavioral response is affected’ [13, p. 84]. It involves two elements [12]: suggestive misleading cues included in the questions asked by the interrogator and negative feedback concerning the quality of the testimony given so far. It is now well known that leading questions and negative feedback given to witnesses can both have a profound negative impact on the quality of witness testimony [14]. Negative feedback is also very important as it is often present in real-life witness interviews [14] and has a negative impact on the accuracy of witnesses’ statements, especially in young persons [15]. Negative feedback does not need to be communicated verbally: it might also be implicit in the repetition of questions [16] or in an interviewer’s unfriendly, unsupportive manner [17].

The standardized procedure for measuring IS (*Gudjonsson Suggestibility Scales*; GSS) [12] begins with reading a short story to the participant. The story is then recalled, and twenty questions are asked, fifteen of which are misleading: e.g. “Did the woman's glasses break in the struggle?”, when in fact no glasses were mentioned in the story. The number of answers indicating acceptance of such misleading premises constitutes Yield: the index of the tendency to give in to suggestive questions. Afterwards, the participant is told that the quality of their testimony was poor, that all questions will be asked again, and they should try harder. All twenty questions are then asked again, and the number of changes in the answer gives the index of the tendency to change answers after negative feedback; this is called Shift (a detailed description of the procedure is given in the “Method” section).

There are not many procedures that have been proven to immunize against misleading questions and negative feedback. One such technique is focused meditation [18]. Although the authors were able to show that this technique is efficient in reducing IS, one might have concerns about the procedure used because groups that used meditation were also informed that they would participate in a relaxation task which had been proven to enhance memory abilities. Thus, the placebo effect as well as the meditation effect could have occurred.

Another technique used in the context of IS is warning [19–21]. In all these studies it proved effective in reducing IS, although it did not eliminate it completely. Moreover, a warning might be dangerous for a witness in the IS paradigm if it is given by friendly (as opposed to hostile) interrogator: in one study [19] participants who received a warning demonstrated an increased number of Shifts in the Friendly condition compared with those who were not warned. In the Abrupt condition (interrogator’s firm and unsupportive demeanor) this pattern was reversed.

The last technique for reducing IS of which we are aware is reinforced self-affirmation (RSA) [22], a technique designed for boosting self-confidence. Compared with the control group, the group with RSA presented significantly lower scores for all measures of IS [23]. RSA will be described in more detail because it was used in the current study.

RSA was primarily based on the assumption that one of the reasons for yielding to suggestion and misinformation is doubting the quality of one’s own memory [10, 24]. This technique aims to boost self-confidence in order to increase the tendency to rely on one’s own memories instead of external cues like misinformation [25]. Self-confidence was chosen as the basis for developing RSA because existing research suggests that it is beneficial in a range of tasks and situations, e.g. leader performance [26], various cognitive competences [27–29], academic achievements [30] or sport [31]. Interestingly, self-confidence has also been shown to predict reliance on oneself as a source of information [32], to reduce susceptibility to social pressure [33], to reduce yield to misleading questions [34] or to blame conformity [35]. Moreover, persons high in self-confidence tend to change their answers in memory tests less frequently than those with low self-confidence [36].

To boost self-confidence, two methods are applied in RSA: self-affirmation and positive feedback. The idea that self-affirmation increases self-confidence was based on existing conceptions and results [37–40]. Similarly, the idea that positive feedback enhances self-confidence was based on existing research [37, 41–43]. Technically, self-affirmation is induced by having participants write down their greatest achievements in life, and positive feedback is achieved by fake positive information about the results on a memory task (see Methods).

The rationale of the efficacy of RSA in reducing vulnerability to mnestic suggestions effect was developed in the context of witness misinformation effect (ME), a paradigm related to IS, although different from it (seminal research: [44]). It consists of the inclusion in witness testimonies of false information from sources other than the given event. It is usually researched by means of a three-stage experimental paradigm in which the participants watch an event take place, afterwards they read a description of this event, which in the experimental group contains some information which is inconsistent with the original event. Finally, they answer questions about the original event, including questions relating to the critical misleading items.

The basic assumption for the RSA as a method for reducing ME was the assumption that there are subjects who at the moment of the final memory test do in fact remember the correct information *and* the misinformation but give a response consistent with the latter simply because they do not trust their own memory [45]. There is evidence confirming that some participants are aware of the inconsistencies between the original and postevent information [10, 11, 46]. It seems that one of the main reasons that participants who in fact do correctly remember the original information yet answer in accordance with the postevent misinformation is their lack of confidence in their own memory [10, 11]. Given this, increasing one’s confidence in the quality of one’s memory should diminish the tendency to doubt it and therefore increase the tendency to answer in accordance with one’s own correct memory, thus reducing the misinformation effect. In accordance with this premise, RSA repeatedly proved effective in reducing yielding to misinformation [22, 25, 45]. In one experiment, evidence was found that RSA was indeed effective mostly among subjects who were aware of the discrepancies between the original and postevent materials [45].

In general, RSA was expected to reduce IS on the basis of the same essential premise: increased confidence in one’s memory. The applicability of this premise in the context of IS can be derived directly from the theory of it. According to the social-psychological model of IS [13] three elements are essential to its mechanism: uncertainty, interpersonal trust, and expectations of success. Uncertainty relates to the fact that the interviewee is usually not sure of the correct answer to a question. Interpersonal trust means that the witness perceives the intentions of the interviewer as genuine. Expectations of success mean the interviewee believes that they should be able to answer the questions correctly.

RSA is related mainly to the first of these premises: uncertainty. The interviewee is often not sure about the correct answer; moreover, they may believe that they have no memory of some particular fact, yet they give in to the leading question because they assume that their own memory is wrong. Also, a participant may be willing to change answers after negative feedback because they believe it and doubt their own memory. Both these tendencies should be reduced if self-confidence regarding memory is increased.

In the present paper, we aimed to replicate and extend the results already obtained [23] in the context of IS. The first form of the RSA procedure, which consists of self-affirmation and positive feedback after a short memory task, might be problematic in the context of GSS as a method for measuring IS because this procedure includes negative feedback itself. Unlike experiments on the misinformation effect, in the GSS a participant receives two opposite kinds of feedback concerning their memory: a positive one in the RSA procedure and a negative one in the GSS procedure. A participant who is first given positive feedback (included in the RSA procedure) and negative feedback (from GSS), both of which concern memory ability, might be confused. Therefore, in the current study we decided not only to replicate the classic RSA procedure, but to extend it by adding different kinds of feedback. More precisely, the memory feedback in the RSA procedure was changed to a sham feedback about the personality traits of a person. In one study [45] some participants–regardless of their answers in computer-based personality tests–received a “diagnosis” stating they were sociable, reliable, helpful to others (the “moral” group), while others received a “diagnosis” that they were assertive and independent in thinking (the “independent self” group). This division resembles “The Big Two”: agency and communion [47, 48] or competence and morality [49]. It was found [45] that RSA in which participants were told that they are very independent in their judgements proved effective in reducing the misinformation effect, contrary to feedback relating to morality.

Another good reason for researching various kinds of feedback is the fact that the form of feedback applied in the original version of RSA assumed overt lying to a subject about the quality of their memory; however, lying to a real witness is difficult due to ethical and legal issues. It would be better if the technique for reducing susceptibility to suggestion was as free of lying as possible. The manipulation used in the presented paper still included fake information (sham positive feedback and sham “diagnosis” of independence), but we aimed to make it less dramatic than faked results on a memory test.

As can be seen, this rationale only refers to the case in which the participants are aware about the discrepancies between information included in the original story and the one suggested by misleading cues included in the questions. If, however misinformation is accepted unconsciously, e.g. through source monitoring errors, RSA would not be effective. We hypothesize that the number of cases in which the participants are in a way aware of the discrepancies is high enough for the RSA to show efficacy.

As the assumption concerning awareness of discrepancies is crucial for the present study, it is worth commenting on. Evidence for the existence of aware participants is present in the case of the misinformation effect [45]. Awareness of discrepancies was not directly analyzed in the present study, but there are results [50] enlightening in this area: it was analyzed whether responses consistent with suggestion were due either to the internalization of misleading information and negative feedback, or simply to compliance. Their participants performed a source identification questionnaire after the GSS had been administered. It contained the questions from the GSS and the participants were asked to indicate where they had originally encountered a specific piece of information: in the ‘story’ or in the ‘questions’ (or they did not know). Suggestible responses in the case of which participants ascribed a given piece of information to the ‘story’ were considered as internalization, and those correctly indicating that the information was in the ‘questions’ were considered compliance. It was found [50] that compliant answers were more frequent than internalization, which means that many subjects gave in to suggestions or to negative feedback even though they knew that the relevant information was not present in the original story. Interestingly, it was also found [50] that internalization was higher in the case of Yield, while compliance prevailed in Shift. In a way, these results are compatible with the present study: they confirm that some participants give in to leading questions and change their answers after negative feedback when they realize that relevant information was present in the questions but not in the story. In sum, it seems that the assumption concerning awareness of discrepancies was confirmed in the case of IS, at least to some extent.

To sum up, the main aims of the experiment presented in this paper were threefold: to replicate the efficacy of RSA related to memory (MemRSA) in reducing IS; to analyze the similar efficacy of RSA in activating independence of judgments (IndRSA); to analyze, for the first time, the efficacy of RSA in combining memory and independence (MemIndRSA).

Following hypotheses were formulated. First, we expected all versions of the RSA to reduce IS. Next, we hypothesized that RSA combining both positive feedbacks: about memory and independence of judgements would be more effective than RSA including one of them. Technically, the following hypotheses were formulated: (1) mean results in the MemRSA group will be lower than in the control group in which no RSA was applied (NoRSA) on Yield (Hypothesis 1a) and Shift (Hypothesis 1b); (2) mean results in the IndRSA group will be lower than in the NoRSA group, on Yield (Hypothesis 2a) and Shift (Hypothesis 2b); (3) mean results in the MemIndRSA group will be lower than in the NoRSA group, on Yield (Hypothesis 3a) and Shift (Hypothesis 3b); (4) mean results in the MemIndRSA group will be lower than in the MemRSA group, on Yield (Hypothesis 4a) and Shift (Hypothesis 4b); and (5) mean results in the MemIndRSA group will be lower than in the IndRSA group, on Yield (Hypothesis 5a) and Shift (Hypothesis 5b).

## Method

Approval for this research was obtained from the Ethical Committee of the Institute of Psychology, Jagiellonian University, Kraków, Poland. Decision nr: KE/01/092018'

### Participants

One hundred and forty-five participants took part in the experiment (90 women, 55 men); their mean age was 26.1 (*SD* = 9.9; range: 15–69). They were recruited *via* advertisements in various media. Consent was obtained and noted by the experimenters; it was verbal in order to ensure the anonymity of the participants. In the case of three minors under 18, verbal consent was obtained from parents. This method of obtaining consent was approved by the Ethical Committee mentioned above. No compensation was given for participating. The size of the sample ensured adequate power (80%) and excellent power (99%) to detect medium effect size (*d* = .5) and a large one (*d* = .8), respectively.

### Materials and procedure

Participants were tested individually. They were invited to an experiment seemingly concerning the ‘psychology of witness testimony’. After having informed them that they could stop participating in the study at any point without providing a reason, participants gave their informed consent to participate in the experiment. To measure IS, version 2 of GSS was used [12]; Polish adaptation: [51]. In this procedure, a narrative is read aloud to participants and they are asked to recall everything they can remember. The story describes a couple saving a boy from having an accident on his bicycle. It includes 178 words and 40 countable ‘chunks’ of information. A 50-minute delay follows, after which participants are asked to recall everything again. The interview phase follows, during which 20 questions are asked by the interviewer, 15 of which are misleading: e.g. ‘Did the couple have a dog or a cat?’, when in fact no pets were mentioned in the story. Giving an answer consistent with the misleading cue is scored one point; in sum, a participant may score from 0 to 15 points on the index of yielding to misleading questions, referred to as Yield. Afterwards, each participant is firmly told that they have made a number of errors and therefore need to go through the questions again, but this time they should try to be more accurate. All 20 questions are then asked again and each clear change in an answer is scored one point, giving the index of the tendency to change answers after negative feedback (Shift), ranging from 0 to 20 points. Yield and Shift may be summed to make the index of total suggestibility.

The experiment was performed by four female students of psychology with appropriate training, strictly following the procedure described in the manual [12]. Each experimenter performed the whole procedure and the whole experiment took place in one room. The experimenters were blind to the purpose of the study. The experimental conditions were varied in random order.

The time delay (about 50 minutes) between the immediate and delayed recall was filled with the RSA procedure and some unrelated questionnaires. The RSA procedure started with the self-affirmation induction, which consisted in the participants writing their greatest life accomplishments. The participants in the control group (NoRSA) described their route from home to the laboratory. The second part of the procedure was different in the four groups. In the NoRSA group, the participants performed some psychological tests without being given any feedback. In the MemRSA group, the participants had to memorize as many nouns as possible from a list of 60 nouns and write them down (5 minutes). They were then given false positive feedback concerning the “quality of their memory”. In the IndRSA group, subjectively perceived independence of judgements was induced via a ‘Computerized Personality Test’ [45]. The computer displayed questions taken from various questionnaires and finally presented the participant with the ‘result’, which stressed that he/she ‘is assertive and independent in thinking’, ‘loves to present their own opinions’ and ‘believes in having their own opinions, not relying on others’. In the MemIndRSA group, procedures concerning both memory and independence of judgements were applied, in the same order for all participants.

After the RSA, a small questionnaire was administered in order to perform the manipulation check; it consisted of five questions relating to independence of judgements answered on a 5-point Likert-like scale, e.g. “I prefer to make decisions myself than to seek advice from others”. To assess the efficacy of the manipulation for increasing confidence concerning memory, a 100mm VAS scale was applied with the instruction, “Please mark with a vertical line on the following line how much you feel at this point that you remember the story well”. The second part of the GSS then took place, in the form of a face-to-face interview, performed by the same interviewer which presented the story, after which the participants were debriefed. Recalling of the story and both rounds of the questions usually took 10–15 minutes. (For the details of the GSS procedure, the content of the story and all the questions, please refer to the manual of this tool [12]).

## Results

In this research, specific *a priori* hypotheses were drawn up concerning differences between mean results in given pairs of groups. Therefore, the analytic strategy recommended for the *a priori* approach was applied: no overall ANOVA was performed and instead analysis of variance in the form of focused comparisons in the form of planned contrasts were calculated [52–54].

At the beginning, age and gender differences across four experimental conditions were analyzed, with insignificant results (age—ANOVA: *F*(3, 141) = 0.08, *p* = 969, η^2^ < .01; gender—chi-square test: *chi*^*2*^(3, *N* = 145) = 3.31, *p* = .346).

### Manipulation check

The NoRSA group (*M* = 59.91, *SD* = 23.04) was compared against the MemRSA (*M* = 70.38, *SD* = 20.81), IndRSA (*M* = 67.57, *SD* = 18.25) and MemIndRSA (*M* = 69.61, *SD* = 21.30) groups. As predicted, memory confidence was significantly higher in the MemRSA group than in the NoRSA group, *F*(1, 141) = 4.51, *p* = .035, ƞ^2^ = .03. The planned comparison between the NoRSA and MemIndRSA groups was near the *alpha* level, *F*(1, 141) = 3.82, *p =* .052, ƞ^2^ = .03), thus one can say that the latter group had higher memory confidence, at the edge of significance. There was no statistical difference between the IndRSA an NoRSA groups in memory confidence, (*F*(1, 141) = 2.41, *p =* .122, ƞ^2^ = .02). In sum, these results confirmed the efficacy of the MemRSA manipulation.

Similar analyses were performed for the IndRSA manipulation. The NoRSA group (*M* = 16.54, *SD* = 3.51) proved to have lower feelings of independence of judgements compared with the IndRSA group (*M =* 18.54, *SD* = 4.37; *F*(1, 141) = 4.14, *p* = .044, ƞ^2^ = .03) and with the MemIndRSA group (*M* = 18.89, *SD* = 4.44; *F*(1, 141) = 5.65, *p* = .019, ƞ^2^ = .04). The NoRSA and MemRSA (*M* = 16.68, *SD* = 4.26) groups did not differ as regards feelings of independence (*F*(1, 141) = 0.02, *p* = 0.892, ƞ^2^ < .01). Thus, the efficacy of the IndRSA manipulation was confirmed.

### Main analyses

Descriptive results concerning the mean result in Yield and Shift are given in Table 1.

**Table 1:** Means (*SD*s, range) [95% confidence intervals] of suggestibility measures in GSS across experimental conditions.

Feedback in RSA	*n*	Yield	Shift
No feedback (NoRSA)	35	6.17 (3.90, 0–15) [4.83, 7.51]	4.49 (2.93, 0–10) [3.48, 5.49]
Memory (MemRSA)	37	4.16 (3.35, 0–11) [3.05, 5.28]	3.03 (2.29, 0–9) [2.26, 3.79]
Independence (IndRSA)	37	6.27 (3.65, 0–15) [5.05, 7.49]	3.00 (3.31, 0–15) [1.90, 4.10]
Memory + Independence (MemIndRSA)	36	4.42 (3.25, 0–12) [3.32, 5.52]	3.11 (2.69, 0–10) [2.20, 4.02]

#### Yield

As stated in hypotheses 1a and 3a, the NoRSA group had significantly higher Yield compared to the MemRSA group (*F*(1, 141) = 5.78, *p* = .017, η^2^ = .04) and to the MemIndRSA group (*F*(1, 141) = 4.35, *p* = .039, η^2^ = .03). However, contrary to hypothesis 2a, there were no significant differences between the NoRSA and IndRSA groups (*F*(1, 141) = 0.01, *p* = .906, η^2^ < .01).

Yield was significantly higher in the IndRSA group compared to the MemRSA group (*F*(1, 141) = 6.55, *p* = .012, η^2^ = .04) and to the MemIndRSA group (*F*(1, 141) = 4.99, *p* = .028, η^2^ = .03), thus confirming hypothesis 5a. There was no significant difference between the MemRSA and the MemIndRSA groups (*F*(1, 141) = 0.09, *p* = .759, η^2^ < .01), leaving hypothesis 4a without confirmation.

#### Shift

In accordance with hypotheses 1b, 2b, and 3b, Shift was significantly higher in the NoRSA group compared to the MemRSA (*F*(1, 141) = 4.77, *p* = .030, η^2^ = .03), IndRSA (*F*(1, 141) = 4.96, *p* = .028, η^2^ = .03) and MemIndRSA (*F*(1, 141) = 4.19, *p* = .042, η^2^ = .03) groups.

No significant differences were found between the MemRSA and IndRSA groups, (*F*(1, 141) < .01, *p* = .967, η^2^ < .01), MemRSA and MemIndRSA (*F*(1, 141) = .02, *p* = .899, η^2^ < .01), or between IndRSA and MemIndRSA (*F*(1, 141) = .03, *p* = .967, η^2^ < .01). Thus, hypotheses 4b and 5b, concerning the superiority of the combination of both methods over each one alone, were not confirmed.

In sum, MemRSA effectively reduced IS both in the case of Yield and Shift, while IndRSA only worked in the case of Shift. Both techniques combined were effective in reducing both Yield and Shift indices.

## Discussion

The aim of this study was to present new empirical results concerning a procedure for inducing resistance to interrogative suggestibility (IS) which aim was to increase self-confidence and feelings that one is independent in their judgements. The results fully confirmed the hypotheses in the case of Shift: MemRSA, IndRSA and MemIndRSA all reduced it. As for Yield, the results confirmed the efficacy of MemRSA and MemIndRSA, but not IndRSA. The results regarding MemRSA were the same as those already obtained [55] where it was effective in the case of both Yield and Shift.

Thus, in the case of Yield, boosting confidence concerning the quality of memory indeed seems to make participants rely on their own memories instead of following the cues in the suggestive questions. Inducing independence of judgements might not have been effective because it taps opinions instead of the quality of a cognitive process like memory. Increasing feelings of independence in opinions may still leave participants with doubts as regards the quality of their memory and therefore make them want to look for cues in the leading questions instead of relying on their own memory. Simply put, participants might believe in their own opinions and views and still be unsure about the quality of their own memory. This may explain why boosting memory confidence regarding the quality of memory was effective in immunizing against misleading questions, while increasing feelings of independence of judgements was not. Different processes seem to be involved in the case of Shift: increased confidence about both memory and independence of judgements may be helpful. If participants are confident in their memory, they do not change their answers easily. In a similar vein, those who believe that they are very independent in their opinions may want to reject the pressure to change answers. In other words, Shift is more related to social influence than Yield. In the case of Yield, no pressure is involved: the interrogator is just asking questions (albeit leading ones). In contrast, for Shift the pressure on the part of the interrogator is apparent: he/she explicitly tells the interviewee to “try to be more accurate this time”. It is possible that boosting participants’ confidence that they are very independent in their judgements is more effective in inducing resistance to social influence than in making someone reject information included in questions only.

Interestingly, in the case of Shift reinforced self-affirmation in both its forms (relating to memory and to independence of judgements) seemed to be able to overcome the *negative* feedback included in the GSS procedure. RSA was applied before the questions from the GSS were asked, so in the case of Yield the participants received just one piece of feedback: the positive feedback included in the RSA. However, *negative* feedback was given afterwards: participants were told that they had made a number of mistakes and that it was necessary to go through the questions again whilst trying to be more accurate. Thus, the participants in the RSA groups received two contradictory pieces of feedback. Despite this, the tendency to change answers was effectively reduced by both forms of RSA (relating to memory and to independence of judgements). This may mean that RSA is more effective than the one-sentence negative feedback given in the GSS. As a result of the competition between these two contradictory types of feedback, in the end self-confidence was still high enough.

The obtained results suggest the superiority of memory feedback over the independence of judgements as regards reducing IS. The combination of these two types of feedback had the same efficacy as memory feedback alone in the case of Yield and Shift, and as independence of judgements in the case of Shift. The only difference that proved significant was the one between MemIndRSA and IndRSA in the case of Yield. Independence of judgements was not helpful in reducing Yield, presumably because of a lack of explicit social pressure in the leading questions (see above). In general, the present results suggest that there is no need to apply both kinds of feedback. This may be an important conclusion for efforts to apply RSA in real situations.

A comment is needed regarding the ‘discrepancy detection’ principle [56, 57] according to which a witness is most likely to incorporate misinformation in their testimony when they do not detect discrepancies between this misinformation and the original event. Indeed, poor discrepancy detection is often given as an explanation of particularly high suggestibility [14]. However, what is important for the present study is that detecting discrepancies by no means *prevents* a subject from giving an answer consistent with misinformation [10, 11]: many people still give in to misinformation despite the fact that they possess all the information necessary to give a correct answer.

In the end, it is worth stressing that given the very large age range (16–69 years), the results obtained are relatively generalizable to the broad population. By no means are the results restricted to the population of students.

### Limitations and future directions

The basic limitation of the present study is related to its poor applicability in real-life forensic settings. In a real witness interview, no one ought to trick a witness, no matter the method or reason. Developing a method based on the ideas presented in this paper that could be used in real forensic settings remains an important task for future research. In particular, a method of delivering positive feedback without overt lying to the witness is warranted.

Secondly, currently nothing is known about the durability of the effects of RSA. In all existing experiments (including the present one), its effects were studied immediately after it. From a practical point of view this may be important. If a psychologist applies a method to improve the quality of testimony, it is desirable that the effects last for a significant time. In the case of RSA, such permanency of effects remains to be studied.

A possible problem may be caused by the fact that the MemRSA and MemIndRSA conditions involved additional memory tasks, whilst the other did not. These tasks could have distorted the participants’ memories for the initial event and could have caused them to remember less than controls. However, this should result in *higher* suggestibility in these conditions, because uncertainty about the original event usually involves greater impact of misinformation [10], which was not the case in the present research.

In two experimental condition group, participants engaged in writing some stories about their self, while in another condition they engage in a memorization task and in the other conditions participants engage in responding to some questionnaires. This is possibly a confounding variable.

An interesting question is whether one of the two techniques constituting RSA alone could have led to comparable results or not. Some existing results suggest a negative answer [58] but they concern the ME, not IS. A research is needed to determine the efficacy of self-confidence or self-affirmation alone in the context of IS.

It should be mentioned that the basic assumption stating that RSA diminishes the tendency to comply with suggestive premises was not tested directly in the present study. The same applies to the assumption that there is some discrepancy detection at all. This would require additional source monitoring testing. Such research is an important future direction, and relevant experiments are already in progress in our laboratory.

Finally, it is always possible that some of the participants discovered the real purpose of the study and acted to confirm its hypotheses. Unfortunately, no post-experimental review was performed to control for this.

## Supporting information

S1 File Raw data. Raw data file.
(CSV)
